# Abnormal brain activity patterns during spatial working memory task in patients with end-stage renal disease on maintenance hemodialysis: a fMRI study

**DOI:** 10.1007/s11682-020-00383-7

**Published:** 2020-09-29

**Authors:** Jinzhuang Huang, Lei Xie, Ruiwei Guo, Jinhong Wang, Jinquan Lin, Zongbo Sun, Shouxing Duan, Zhirong Lin, Hui Li, Shuhua Ma

**Affiliations:** 1 Department of Radiology, the First Affiliated Hospital of Shantou University Medical College, Shantou, 515041 China; 2 Shantou University Medical College, Shantou, 515041 Guangdong China; 3 Guangdong Key Laboratory of Medical Molecular Imaging, Shantou, 515041 China; 4 Department of Ultrasound, the First Affiliated Hospital of Shantou University Medical College, Shantou, 515041 China; 5 Department of Pediatric Surgery, the First Affiliated Hospital of Shantou University Medical College, Shantou, 515041 China; 6 Mental Health Center, Shantou University Medical College, Shantou, 515000 China

**Keywords:** Spatial working memory, End-stage renal disease, Hemodialysis, Brain activity patterns, Functional magnetic resonance imaging

## Abstract

Hemodialysis (HD) is associated with cognitive impairment in patients with end-stage renal disease (ESRD). However, the neural mechanism of spatial working memory (SWM) impairment in HD-ESRD patients remains unclear. We investigated the abnormal alterations in SWM-associated brain activity patterns in HD-ESRD patients using blood oxygen level-dependent functional magnetic resonance imaging (BOLD-fMRI) technique during n-back tasks. Twenty-two HD-ESRD patients and 22 well-matched controls underwent an fMRI scan while undergoing a three-load n-back tasks with different difficulty levels. Cognitive and mental states were assessed using a battery of neuropsychologic tests. The HD-ESRD patients exhibited worse memory abilities than controls. Compared with the control group, the HD-ESRD patient group showed lower accuracy and longer response time under the n-back tasks, especially in the 2-back task. The patterns of brain activation changed under different working memory loads in the HD-ESRD patients, showing decreased activity in the right medial frontal gyrus and inferior frontal gyrus under 0-back and 1-back task, while more decreased activation in the bilateral frontal cortex, parietal lobule, anterior/posterior cingulate cortex and insula cortex under 2-back task. With the increase of task difficulty, the activation degree of the frontal and parietal cortex decreased. More importantly, we found that lower activation in frontal cortex and parietal lobule was associated with worse cognitive function in the HD-ESRD patients. These results demonstrate that the abnormal brain activity patterns of frontal cortex and parietal lobule may reflect the neural mediation of SWM impairment.

## Introduction

Cognitive impairment is common in patients with chronic kidney disease (Pi et al. 2016), especially those with end-stage renal disease (ESRD) who are on hemodialysis (HD) (Chen et al. 2015). A previous study reported a 30–60% prevalence of cognitive impairment in HD patients, which is twice as high as that in healthy controls (Bugnicourt et al. 2013). There is growing evidence of cognitive deficits in HD-ESRD patients, including impaired attention, cognitive slowing, impaired executive function, disorientation, memory and language impairment (Hermann et al. 2014; Murray et al. 2006; Murray 2008). This may affect their later treatment, such as dietary adjustments and drug adherence. Davey et al. reported that change in renal functioning over time is related to changes observed in global cognitive ability, verbal episodic memory and abstract reasoning (Davey et al. 2013). However, the neural mechanism of cognitive dysfunction in HD-ESRD patients is still unclear and often neglected in clinical practice. Therefore, it is important to study the pattern of cognitive dysfunction in HD-ESRD patients and clarify its exact neural mechanism for improving prognosis.

Working memory is widely recognized as the basis of many advanced cognitive functions and is defined as a component of short-term memory, including verbal, object, and spatial working memory (SWM) that processes different types of information (Goldman-Rakic 1996). Working memory has been widely considered as the foundation of many higher cognitive functions, such as learning, language understanding, reasoning, and judgment, and it has also been closely linked with other cognitive behaviors (D’Esposito et al. 1995). Functional magnetic resonance imaging (fMRI) allows the examination of neural substrates associated with early cognitive changes, in ESRD patients undergoing dialysis, before clinically significant symptoms of cognitive impairment become apparent (Chen et al. 2015; Luo et al. 2016). Compared with healthy or chronic kidney disease (CKD) controls, neuroimaging abnormalities, such as white matter hyperintensity (WMH), brain atrophy, and dominant or recessive cerebral infarction, are prevalent in brain MRIs of HD patients (Fazekas et al. 1995; Drew et al. 2013). A recent TBSS study by Yin et al. (Yin et al. 2018) investigated the microstructural changes of WM over the whole brain in patients with ESRD, and found that damage to the thalamic radiation and corona radiata may affect cognitive function in ESRD patients. More importantly they proposed that the reduced integrity of ATR may tend to affect the working memory, whereas damage to the corona radiata may involve impaired executive function seen in ESRD patients. The accumulation of serum creatinine and blood urea nitrogen may contribute to the WM impairment (Radić et al. 2010; Zheng et al. 2014). However, few studies have examined the effects of HD on cognitive function or the incidence of structural and functional neuroimaging abnormalities in ESRD patients, and most of these studies detect spontaneous neural activity by resting-state functional MRI (rs-fMRI) (Prohovnik et al. 2007; Radic et al. 2011; Wolfgram et al. 2015). A study of kidney disease and cognitive function reported that CKD is related to a wide range of deficits in cognitive function, including verbal and visual memory and organization, and components of executive functioning and fluid intellect (Elias et al. 2013). Spatial working memory, which temporarily stores spatial information, is an independent component of the Baddeley working memory model with limited capacity and specific neural basis, and the neural pathways processing spatial information are different from verbal and object working memory (Baddeley 1992; Jonides et al. 1993; Smith et al. 1996). So, it is important to study whether spatial working memory is impaired in HD-ESRD patients. To our knowledge, there are no publications of data about brain activity alterations in HD-ESRD patients under different working memory loads.

Therefore, the goal of our study was to prospectively examine alterations in SWM-associated brain activation of HD-ESRD patients by using the BOLD-fMRI technique during a block-designed n-back task, and to determine whether altered patterns of brain activation are associated with exacerbated cognitive decline. We hypothesized that HD-ESRD patients have poor cognitive behavior and abnormal brain functional activities during the n-back task, and the abnormal brain activity patterns of frontal cortex and parietal lobule may lead to SWM impairment.

## Methods

### Participants

Twenty-eight HD-ESRD patients were recruited from the Hemodialysis Department of the First Affiliated Hospital of Shantou University Medical College and Shantou Chaonan Minsheng Hospital, Shantou City, Guangdong Province, China, between September 2015 and October 2018. Among the 28 HD-ESRD patients, 6 were excluded, 5 due to severe mental disorder or cerebral infarction, and 1 due to excessive head motion (see data preprocessing). Finally, 22 HD-ESRD patients and 22 healthy controls, well-matched in terms of sex, age, and years of education (all *P*s > 0.05), were enrolled in the present study. All subjects were right-handed and had normal vision or good corrected visual acuity. Demographic results of the HD-ESRD patients and controls are shown in Table 1.

**Table 1:** Demographic, clinical characteristics, and neuropsychological tests results of HD-ESRD patients and controls

**Variables**	**Controls (n = 22)**	**HD-ESRD (*n*** **= 22)**	***P*** **value**
Age (year)	34.59 ± 6.48	33.33 ± 6.41	0.641
Gender (male: female)	12:10	12:10	1.000
Education(year)	11.51 ± 4.68	12.54 ± 2.53	0.233
Creatinine (μmol/L)	–	851.75 ± 257.56	–
Urea (mmol/L)	–	19.50 ± 7.83	–
Hb (g/dL)	–	96.22 ± 21.54	–
Disease duration (m)	–	24.62 ± 4.91	–
Dialysis duration (m)	–	6.71 ± 2.85	–
MoCA (score)	28.67 ± 0.65(	23.67 ± 3.26	0.007
WMS (score)	103.00 ± 5.25	96.90 ± 4.68	0.002
MMSE (score)	29.18 ± 0.58	27.85 ± 0.63	0.024
HAD-M (score)	6.85 ± 0.55	6.45 ± 1.33	0.280
SAS (score)	28.85 ± 6.31	30.25 ± 7.65	0.095

All quantitative data are expressed as mean ± SD; qualitative data (Gender) is expressed in terms of quantity. HD, hemodialysis patients; ESRD, end-stage renal disease; Hb, hemoglobin; MoCA, Montreal Cognitive Assessment; WMS, Wechsler Memory Scale; MMSE, Mini-Mental State Exam; HAD-M, Hamilton Depression Rating Scale; SAS, Self-Rating Anxiety Scale.

Inclusion criteria for the HD-ESRD patients in this study were as follows: (a) a diagnosis of ESRD according to the K/DOQI grading of CKD; (b) between the ages of 20 and 50, irrespective of gender; (c) had undergone maintenance hemodialysis for at least 3 months; (d) No acute renal failure (ARF), and no history of kidney transplantation; (e) Not taken any psychotropic drugs for at least 1 year; (f) normal vision and right-handedness. Exclusion criteria were: (a) a history of neuropsychiatric diseases, head trauma, stroke, ear surgery or addiction; (b) MRI contraindications; (c) had a head motion of more than 2 mm or 2° during MR scanning.

This study was approved by Medical Ethics Committee of Shantou University Medical College. All subjects gave written informed consent prior to the study.

### Laboratory tests

All HD-ESRD patients underwent blood biochemical tests, including hemoglobin, serum creatinine and urea conditions, within 24 h before MR scanning. The international guidelines, published in 2013 by Kidney Disease Improving Global Outcomes (KDIGO), recommend the use of several techniques to assess renal function. Glomerular filtration rate (GFR) is one of the most commonly used measures of global renal function and is defined as the clearance by filtration of a marker from the plasma by the kidneys, and the two most popular markers of kidney function used clinically are serum creatinine and blood urea nitrogen (KDIGO CKD Work Group, 2013). Collection and testing were carried out by two professional doctors from the Clinical Laboratory Department of the First Affiliated Hospital of Shantou University Medical College. None of the healthy controls underwent the above laboratory tests and therefore no data were available for analysis of healthy subjects.

### Neuropsychological tests

Cognitive and mental states were assessed in all subjects using a battery of neuropsychological tests including the Montreal Cognitive Assessment (MoCA), Wechsler Memory Scale (WMS), Mini Mental State Exam (MMSE), Hamilton Depression Rating Scale (HAM-D) and Self-Rating Anxiety scale (SAS) (Gong et al. 1989; Kimmel et al. 2007; Nasreddine et al. 2005; Theofilou 2011; Wang et al. 2010; Zung 1971). The above neuropsychological tests were performed by two experienced neuropsychiatrists within 1 h after MR scanning.

The Montreal Cognitive Assessment (MoCA) was designed to evaluate cognitive and memory function. The MoCA Chinese-revised (MoCA-CR) includes visuospatial/executive, naming, memory, attention, language, abstraction, delayed recall, and orientation, using a total of eight subtests, and provides an overall score of 30, using a standard for evaluation according to Wang et al. (Wang et al. 2010), which involves calculating overall scores and correcting for the education bias (such as less than 12 years of education increases the score by 1 point); 26 points or greater is considered normal cognitive function, < 26 points is considered mild-cognitive dysfunction; MoCA score ≤ 19 points indicates dementia.

The Wechsler Memory Scale (WMS) was designed to evaluate memory functions. The WMS Chinese-revised (WMS-CR) includes personal experience, orientation, mental control, figural memory, visual recognition, visual reproduction, associative learning, a touch test, understanding memory, and transient memory (numeric span) in a total of 10 subtests and an overall“memory quotient”.

The MMSE Chinese-revised (MMSE-CR) test was administered to all subjects to evaluate the individual’s general cognitive states, and used a scale of 0–30. Those scoring 26 points or greater were considered as having normal cognitive function, and <26 points as possibly having mild cognitive dysfunction, and an MMSE score <23 was used for excluding possible dementia.

Because depression is important in research focused on cognitive function and is more common in HD-ESRD patients, the HAM-D scale was also administered, with a score greater than 14 indicating positive anxiety, a score greater than 7 indicating anxiety, and a score less than 6 indicates no anxiety.

SAS is considered a sensitive and ecologically valid measure of subjective anxiety and depression. According to the results of the Chinese norm, the standard score of SAS is 50, from which 50–59 indicates mild anxiety, 60–69 indicates moderate anxiety, and above 70 indicates severe anxiety.

### Spatial working memory task paradigm

**Fig. 1:**
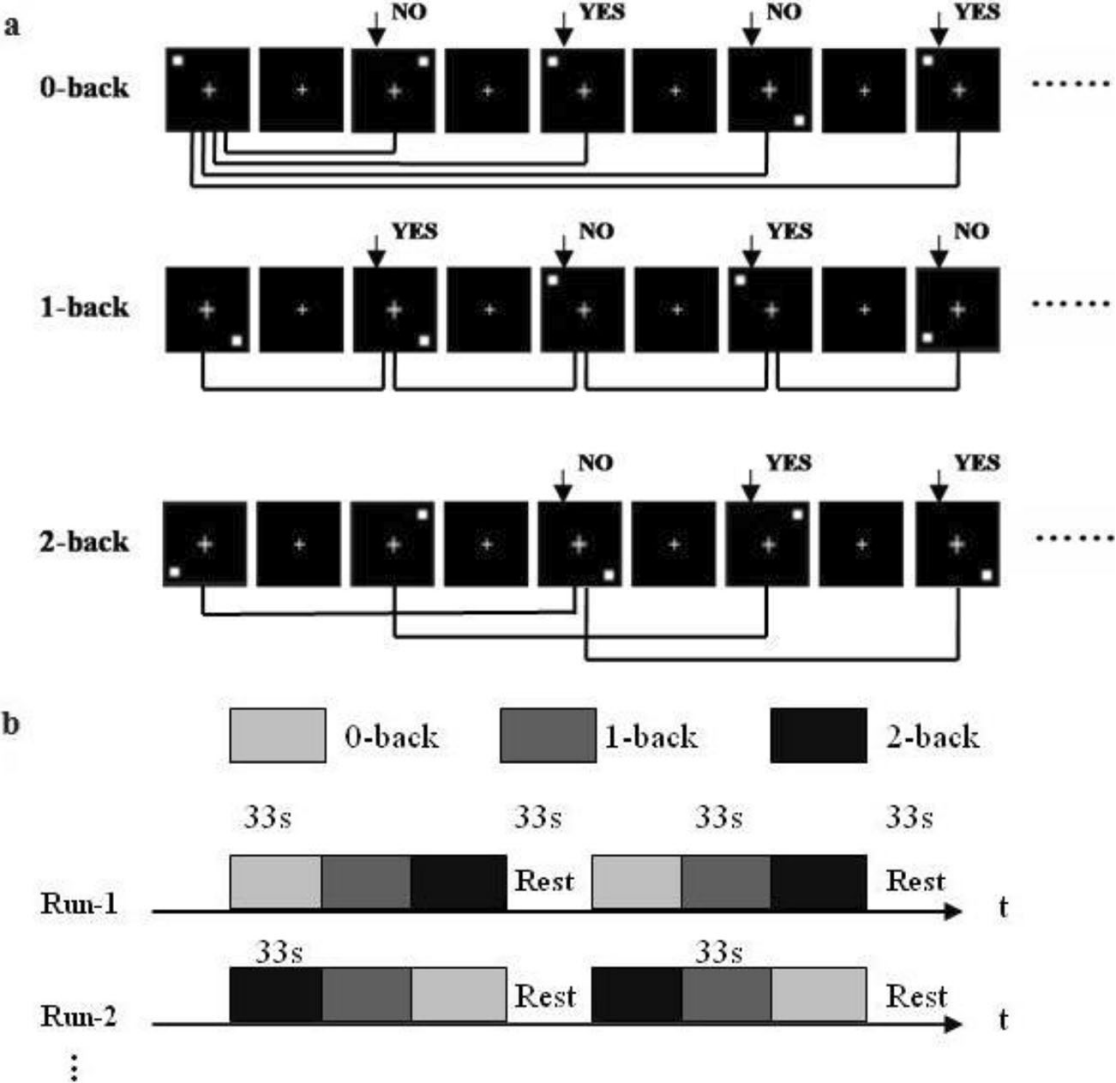
Spatial working memory task paradigm. (a) A blocked design n-back task with three task conditions (0-, 1-, and 2- back). (b) All subjects underwent four fMRI scans (Run-1 -2, −3, −4, with Run-3 the same as Run-1, and Run-4 the same as Run-2), each fMRI scan contained two epochs, each epoch comprised four blocks, 0-, 1-, 2- back condition task block, followed by a baseline control block

As in our previous studies (Huang et al. 2016; Liao et al. 2012; Yin et al. 2012), the spatial working memory of all subjects was assessed using a block-designed n-back task with three load conditions (0-, 1-, and 2- back) during fMRI scanning (Fig. 1a). White box (white on a black background) was presented to participants in pseudorandom order. In 0-back task, all subjects needed to determine whether the current position of the white box is the same as that first presented, while in 1- and 2- back task, to determine whether the current position of the white box is the same as that presented in the previous one or two stimuli. Participants answered by pressing the response button (right button for “YES”, and left button for “NO”) with both hands thumb.

All the subjects underwent four fMRI scans (Run-1 -2, −3, −4, Run-3 was the same as Run-1, and Run-4 was the same as Run-2), each fMRI scan contained two epochs, each epoch comprised four blocks, three load conditions (0-, 1-, and 2- back) blocks, followed by a resting-state (“Rest”) block as baseline control (Fig. 1b). Each task block contained a 3 s task prompt and a 30 s task stimulus (10 stimuli, each displayed for 2 s and followed by a fixed crossover for 1 s). The “Rest” block lasted 33 s with the fixed crossover. Thus, each fMRI scan took 264 s.

Participants were well trained and practiced before the fMRI scan to ensure they understood the requirements of our task. The n-back task paradigm was presented using E-prime 2.0 software (https://pstnet.com/products/e-prime) on a computer that was compatible and synchronized with the MR device. The response accuracy and reaction times during task completion could be recorded simultaneously, along with recording the numbers of correct button presses in response to targets (= hits), the numbers of incorrect button presses in response to non-targets (= commission errors or false alarms), and the numbers of no button presses in response to targets (= omission errors or misses).

### MR imaging data acquisition

MR imaging data were collected using a GE 1.5 Tesla magnetic resonance scanner from the Department of Radiology, the First Affiliated Hospital of Shantou University Medical College.

fMRI images during the spatial working memory task were acquired using a gradient-echo echo-planar imaging (GRE-EPI) sequence, with the following parameters: TR = 2000 ms, TE = 45 ms, field of view (FOV) of 230 mm × 230 mm, matrix of 64 × 64, flip angle of 90°, slice thickness = 6 mm, 1 mm intersection gap, and 20 axial slices. Each fMRI scan lasted 264 s and obtained 132 image volumes. Three-dimensional (3D) data of the whole brain was acquired using fast low-angle radio frequency pulse sequence: TR = 30 ms, TE = 3.0 ms, FOV = 250 × 250 mm, matrix = 256 × 256, slice thickness = 1.3 mm, no gap, and 120 slices. The anterior-posterior commissure line was selected for the scan baseline.

### fMRI data processing and analysis

fMRI data were preprocessed and analyzed using AFNI software (Analysis of Functional NeuroImages, http://afni.nimh.nih.gov/afni/). Before fMRI data preprocessing, the first 10 volumes were removed, then slice timing correction was processed to remove any linear drift, motion correction (head motion more than 2 mm or 2° after motion correction was excluded from further analysis), spatial normalization to standard coordinates of the Tallairach and Tournoux atlas, and smoothed with a 6 mm full-width half-maximum (FWHM) Gaussian filter.

In fMRI data for individual and group analysis, according to the direct comparison between the three loading conditions (0-, 1-, 2- back) blocks and the “Rest” baseline control block, correlation analysis was performed to generate brain activation maps for each group (false discovery rate (FDR) criterion, *P* < 0.05, cluster size ≥20 voxels), and further address the brain regions of interest (ROIs). Repeated analysis of variance was used for inter- and intra-group analysis. The average BOLD response amplitude of each subject’s ROIs in the three n-back conditions was calculated and analyzed to determine whether there was a load effect on each ROI. As Gajewski et al. (Gajewski et al. 2018) reported that working memory declines with age, in this study the brain activation differences between HD-ESRD patients and control group were tested using nonparametric permutation testing, and were corrected for age, body weight, and behavioral symptoms. Data were corrected for multiple comparisons using a threshold-free cluster enhancement, which allows the identification of clusters of significant voxels without having to define them in a binary way, as well as family-wise error, using a final FDR correction, *P* < 0.05, cluster size ≥20 voxels. If a statistical difference was present, post hoc t-tests were performed to detect the inter-group difference.

### Statistical analysis

The independent two samples *t*-test was used to compare the demographic data (age, sex and level of education), laboratory and neuropsychologic test data, the performance accuracy and reaction time of n-back task performance. General Linear Model (GLM) analysis was used to evaluate the load effect of n-back task performance of each ROI. Pearson correlation analysis were performed to investigate the relationship between the percentage of BOLD signal change in the frontal cortex and parietal lobule, and the laboratory results, neuropsychological tests, and the performance accuracy and reaction time of n-back task performance in HD-ESRD patients.

SPSS statistics software package (Windows version 20.0, IBM) was used for statistical analysis. A threshold *P* < 0.05 was considered statistically significant. All quantitative data were expressed as the mean ± standard deviation (SD).

## Results

### Demographic, laboratory and neuropsychologic results

There were no significant differences in age (*P* = 0.641), gender (*P* = 1.000), and education duration (*P* = 0.233) between HD-ESRD patients and control group. MoCA and WMS test results were significantly different between the two groups (Table 1). Cognitive function was assessed using MoCA for all subjects (Table 2), resulting in HD-ESRD patients showing poorer total scores for MoCA compared with controls (HD-ESRD patients: 23.67 ± 3.26, Controls: 28.67 ± 0.65, *P* < 0.01), with statistically significant differences between the two groups on abstractness and memory tests (*P*s < 0.05), and visuospatial/executive function, attention, and language tests (*P*s < 0.01), but no significant differences were observed for naming and orientation (*P*s > 0.05). WMS test was used to assess memory function for all subjects (Table 3), HD-ESRD patients had poorer total scores compared with the controls (HD-ESRD patients: 96.9 ± 4.68, Controls: 103.0 ± 5.25, P < 0.01), but no significant differences in personal experience, orientation, or 1 → 100 subtest of mental control and figural memory (*P*s > 0.05), while for other subtests there were statistically significant differences between the two groups, especially for associative learning and memory quotient (*P*s < 0.01). In terms of cognitive performance, the HD-ESRD group showed slightly lower MMSE scores than healthy controls (HD-ESRD patients: 27.85 ± 0.63, Controls: 29.18 ± 0.58, *P* < 0.05). No significant differences were found in HAD-M (HD-ESRD patients: 6.85 ± 0.55, Controls: 6.45 ± 1.33, *P* > 0.05) and SAS test (HD-ESRD patients: 28.85 ± 6.31, Controls: 30.25 ± 7.65, *P* > 0.05) (Table 1).

**Table 2:** MoCA performance of HD-ESRD patients and controls

**Variables**	**Controls (*n*** **= 22)**	**HD-ESRD (n = 22)**	***P*** **value**
Visuospatial/Executive	4.83 ± 0.39	3.83 ± 0.94	0.008
Naming	3.00 ± 0.00	2.75 ± 0.62	0.328
Attention	4.83 ± 0.39	2.92 ± 1.83	0.005
Language	5.92 ± 0.29	5.33 ± 0.89	0.009
Abstraction	2.42 ± 0.67	0.92 ± 0.67	0.015
Memory	1.75 ± 0.62	1.17 ± 0.58	0.033
Orientation	5.83 ± 0.39	5.73 ± 0.44	0.734
Total	28.67 ± 0.65	23.67 ± 3.26	0.007

Data are expressed as mean ± SD.

**Table 3:** WMS Performance of the HD-ESRD patients and controls

**Variables**		**Controls (n = 22)**	**HD-ESRD (n = 22)**	***P*** **-value**
Personal experience		4.90 ± 0.29	4.74 ± 0.45	0.740
Orientation		5.73 ± 0.35	5.83 ± 0.39	0.930
Mental control	1 → 100	11.08 ± 1.08	10.68 ± 0.94	0.870
	100 → 1	10.08 ± 1.08	8.67 ± 0.89	0.035
	Accumulation	10.57 ± 0.82	10.42 ± 0.33	0.880
Figural memory		10.60 ± 1.31	10.10 ± 1.13	0.830
Visual recognition		9.87 ± 0.50	8.45 ± 1.45	0.025
Visual reproduction		10.43 ± 1.82	8.35 ± 1.39	0.018
Associative learning		10.68 ± 1.83	8.16 ± 1.75	0.004
Touch test		9.33 ± 1.20	8.00 ± 1.30	0.045
Understanding memory		9.90 ± 1.51	7.95 ± 1.30	0.028
Numeric span		10.05 ± 1.08	8.90 ± 0.79	0.032
Memory quotient		103.0 ± 5.25	96.90 ± 4.68	0.002

Data are expressed as mean ± SD.

### Performance accuracy and reaction times of n-back task

Performance accuracy of the n-back task refers to the percentage of correctly reported stimuli in response to targets in the total number of stimuli (excluding the numbers of no button presses in response to targets) to be recalled in certain n-back conditions. Reaction time refers to the time it takes to press the button when the stimuli are played. In this study, the performance accuracies of all subjects were over 80%, indicating that every participant tried their best to complete the task and did not use other programs such as giving up part of the tasks in the experiment, thus eliminating the impact of poor performance. The results were shown in Table 4. Analysis within the groups revealed that as the memory task difficulty levels increased from 0-back to 2-back, the performance accuracies of the two groups decreased (presented a decreasing trend, *P* < 0.05), and the reaction times were prolonged (presented an increasing trend, *P* < 0.05), thus indicating that there was no speed-accuracy trade-off effect. Intergroup analysis showed that, compared with the control group, the HD-ESRD patient group showed lower accuracy and longer response time under the three n-back tasks, the difference between the two groups was statistically significant in the 2-back load task (*P* < 0.05), but not in the 0-back and 1-back task (*P*s > 0.05).

**Table 4:** Performance accuracy and reaction time of n-back task for HD-ESRD patients and controls

**Variables**	**Controls (*n*** **= 22)**	**HD-ESRD (n = 22)**	***P*** **value**
Performance Accuracy (%)			
0- back	99.68 ± 0.05	97.35 ± 0.02	0.476
1- back	97.81 ± 0.03	95.40 ± 0.05	0.258
2- back	93.68 ± 0.06	82.44 ± 0.04	0.030
Reaction Time (ms)			
0- back	658 ± 95	707 ± 74	0.750
1- back	770 ± 113	844 ± 105	0.812
2- back	911 ± 191	1574 ± 180	0.002

Data are expressed as mean ± SD.

### Common brain activity patterns of SWM

**Fig. 2:**
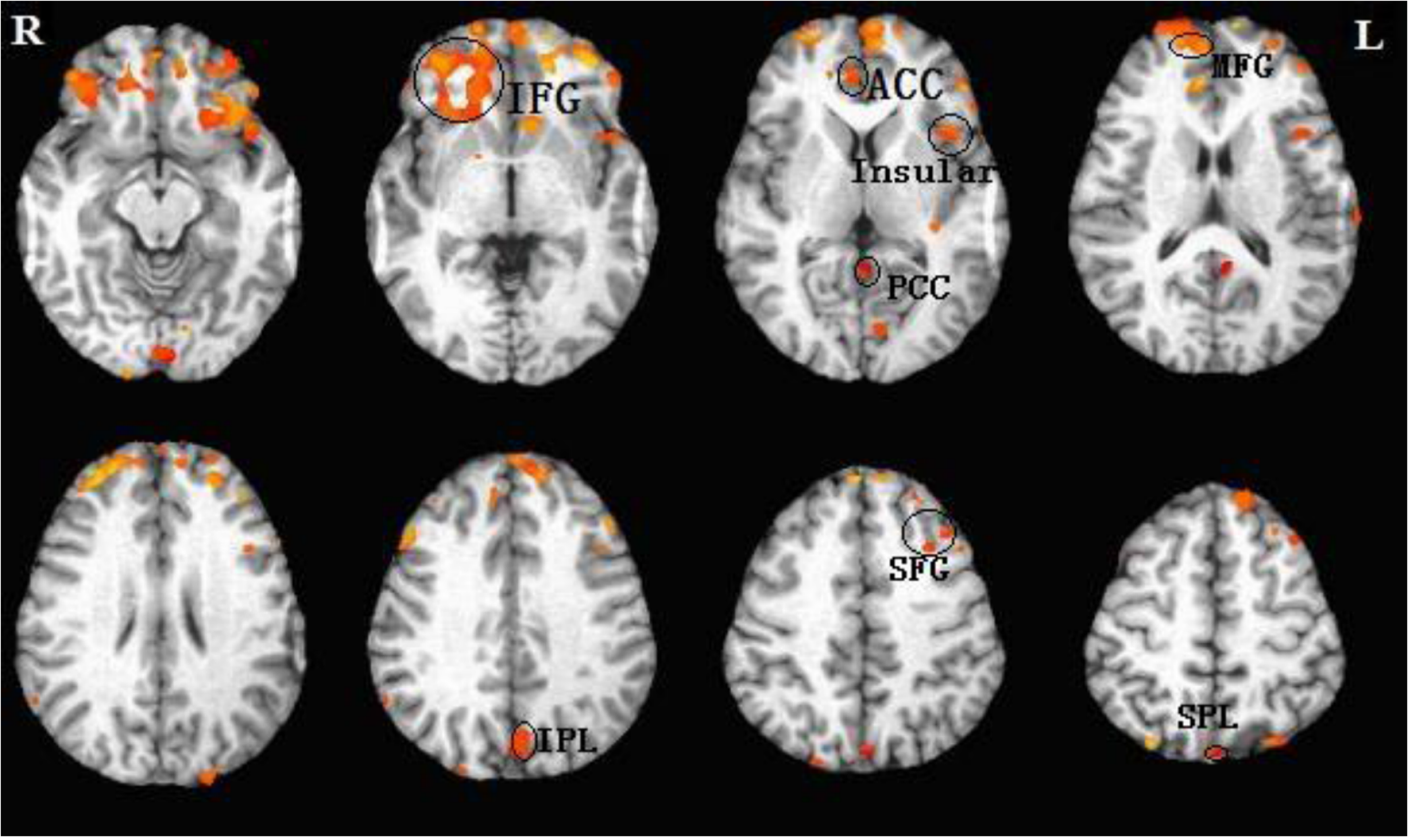
Regions of interest (ROIs) of spatial working memory. fMRI examination revealed common brain activity patterns during the n-back task in both the HD-ESRD patients and control group, including the SFG/MFG/IFG, SPL/IPL, ACC/PCC and insula cortex, with right hemispheric dominance. These brain regions were consistent with those previously reported in studies of working memory so we defined these common brain activity regions as regions of interest (ROIs) of spatial working memory (SWM). L, left hemisphere; R, right hemisphere; SFG, superior frontal gyrus; MFG, middle frontal gyrus; IFG; inferior frontal gyrus; SPL/IPL, superior/inferior parietal lobule; ACC, anterior cingulate cortex; PCC, posterior cingulate cortex

Correlation analysis was performed to generate brain activation maps for each group, and further address the brain regions of interest (ROIs). The average BOLD response amplitude of each subject’s ROIs in the three n-back conditions was calculated, and showed common brain activity patterns during the n-back task in both the HD-ESRD patients and control group, including the superior/middle/ inferior frontal gyrus (SFG/MFG/IFG), superior/inferior parietal lobule (SPL/IPL), anterior/posterior cingulate cortex (ACC/PCC) and insula cortex, with right hemispheric dominance. We defined these common brain activity regions as regions of interest (ROIs) of SWM (Fig. 2), which are also reported in previous studies on working memory using n-back tasks (Huang et al. 2016; Liao et al. 2012; Yin et al. 2012).

### BOLD signal changes of n-back task

**Fig. 3:**
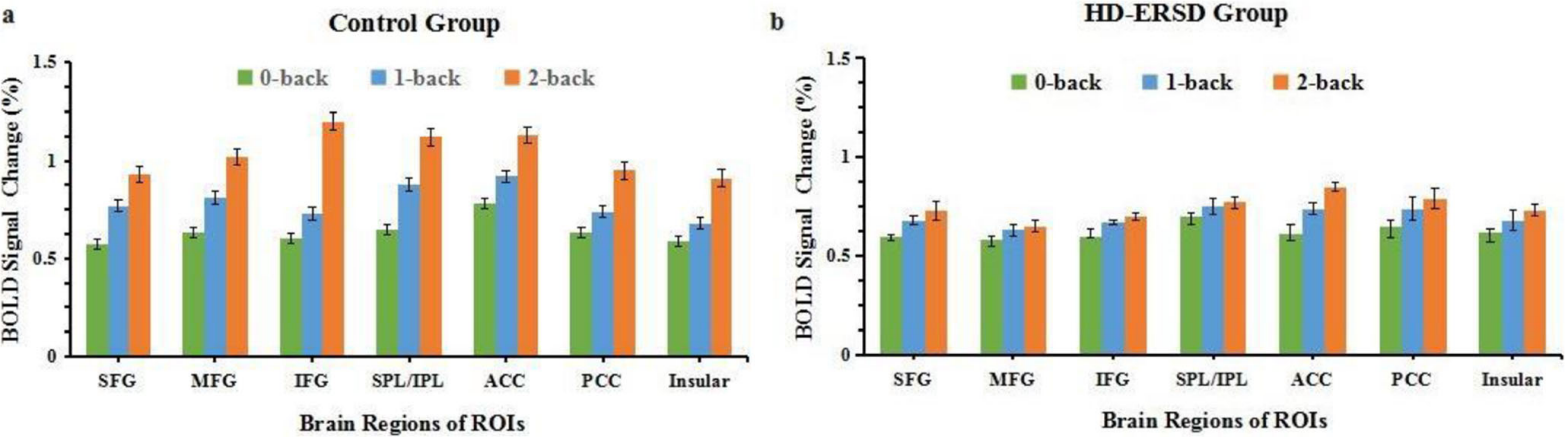
BOLD signal changes of ROIs for the HD-ESRD group and control group under three different n-back task loads. SFG, superior frontal gyrus; MFG, middle frontal gyrus; IFG; inferior frontal gyrus; SPL/IPL, superior/inferior parietal lobule; ACC, anterior cingulate cortex; PCC, posterior cingulate cortex

The BOLD signal responses of the above SWM-related ROIs changed with different n-back task loads. As the memory load increased, more brain regions were activated, and the intensity of these brain regions increased gradually (Fig. 3a,b). In the control group, all ROIs exhibited a task load effect, whereas for the HD-ESRD patients, although some ROIs were activated under three task loads, only the SFG, ACC, PCC and insula cortex exhibited a task load effect, the MFG, IFG and SPL/IPL had no task load effect. In addition, those load effects were more significant under 2- back task load.

### Group activity differences between the three task loads

**Fig. 4:**
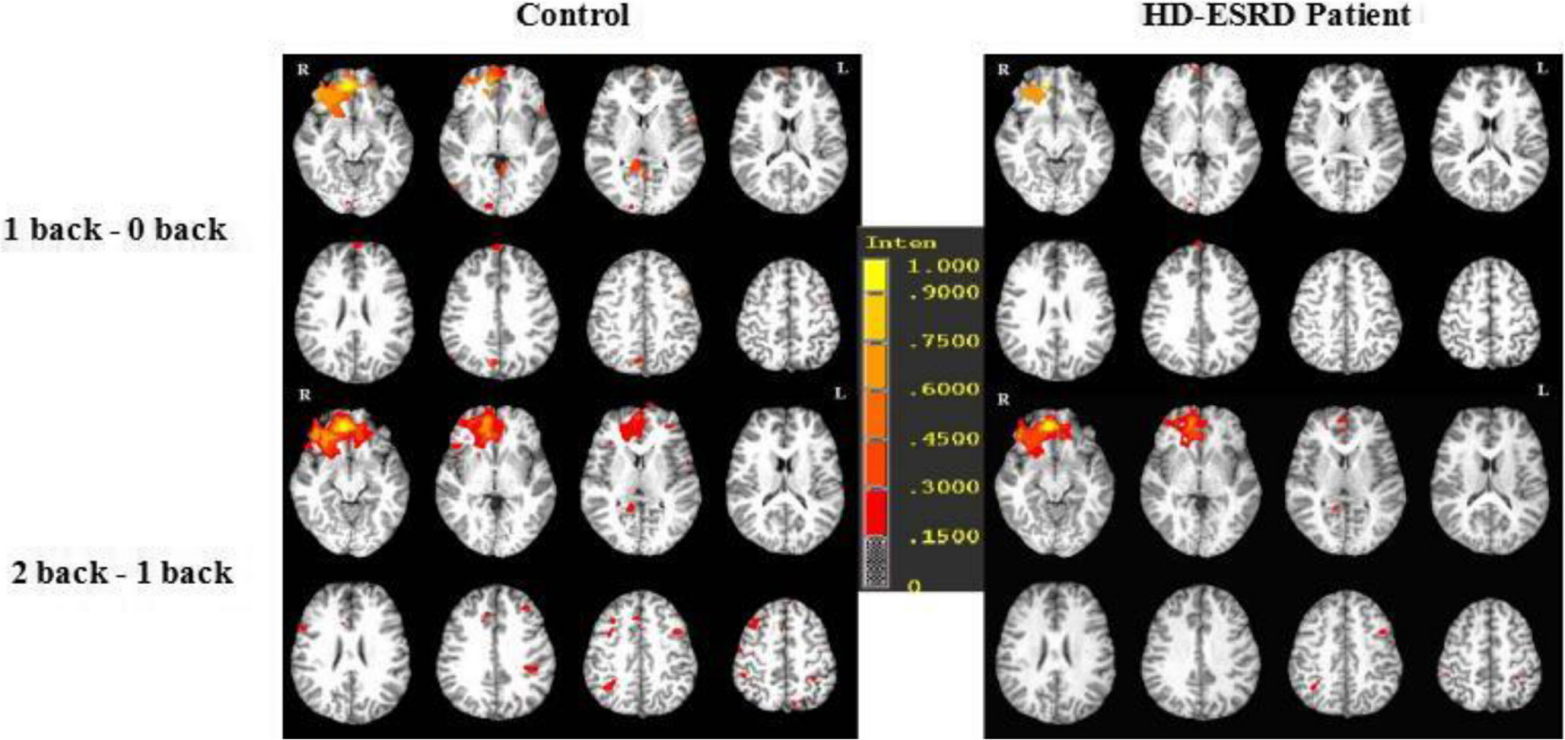
Brain activation differences between the three task loads (1-back vs. 0-back condition, and 2-back vs. 1-back condition) in HD-ESRD patients and control group

**Fig. 5:**
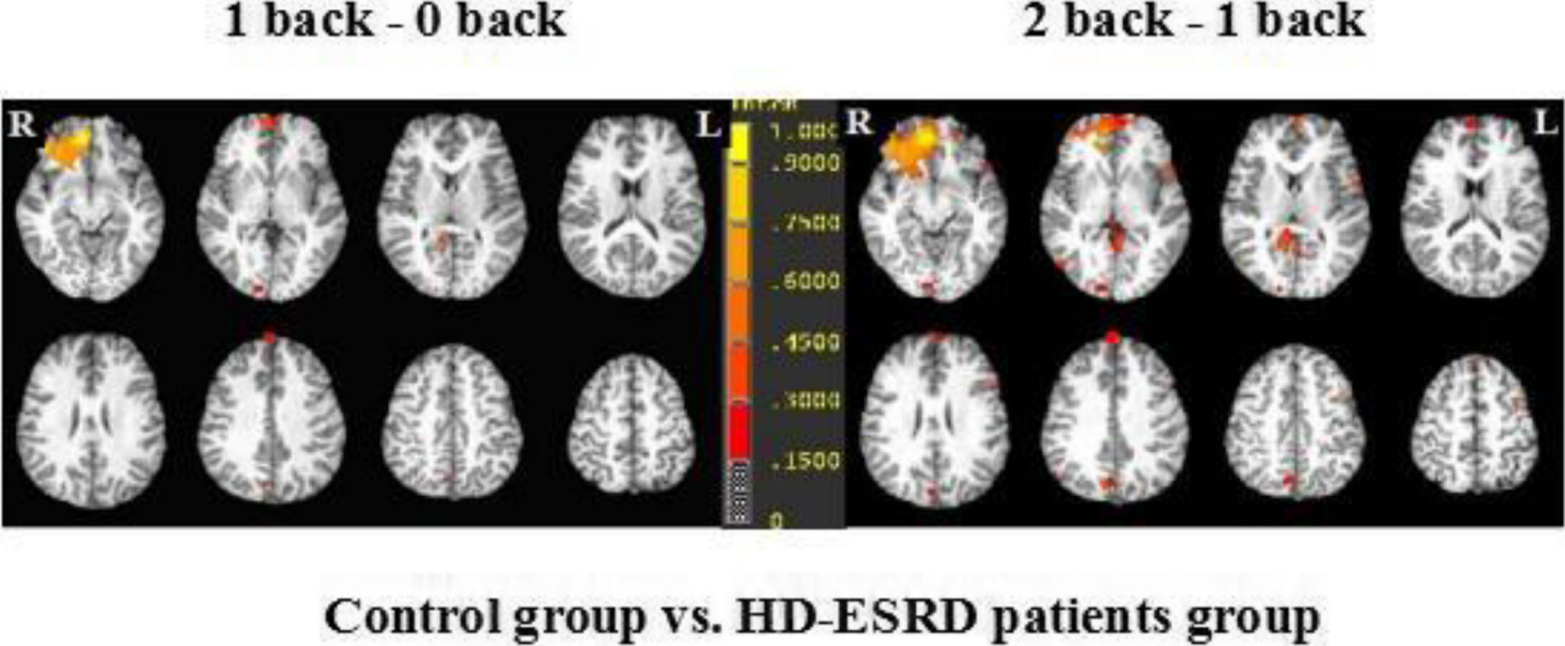
Group differences (Control group vs. HD-ESRD patients group) of the three conditions (1-back vs. 0-back condition, and 2-back vs. 1- backcondition)

We compared the brain activation differences between three different tasks (1-back vs. 0-back condition, and 2-back vs. 1-back condition) in HD-ESRD patients and control group (Fig. 4), and also the group differences (control group vs. HD-ESRD patients group) of the three conditions (1- back vs. 0- back condition, and 2-back vs. 1-back condition) (Fig. 5). The results showed that in the 1-back vs. 0-back comparison, the degree of activation of the right MFG and IFG, right IPL, right ACC and PCC of HD-ESRD patients was lower than that of the controls, while in the 2-back vs.1-back comparison, compared with the control group, the degree of activation of the bilateral SFG, MFG, IFG, SPL/IPL and ACC of HD-ESRD patients was decreased .

### Comparison of brain activity between the two groups

Comparison of brain activity of the n-back task between the two groups indicated that more brain regions were activated, and the intensity of most SWM-related brain regions in the control group was greater than that in the HD-ESRD patients, especially under the 2- back task. We also found that the right MFG and IFG displayed lower activation in the HD-ESRD patients when comparing the 0-back vs. 0-back task between the two groups (Control Group > HD-ESRD Group), while for 1-back vs. 1-back task, the bilateral SFG and MFG, righe IFG, IPL and ACC, PCC and the left insula showed lower activation in the HD-ESRD patients, and for 2- back vs. 2- back task, the bilateral SFG, MFG IFG, SPL/IPL and ACC, PCC and the insula showed less activation in the HD-ERSD patients. In addition, we found that the bilateral parahippocampal cortex and the right precuneus were activated in HD-ESRD patients comparing of the 2- back vs. 2- back task (Table 5).

**Table 5:** Regional differences in brain activation during spatial working memory processing (three loads n-back task) in HD-ESRD patients versus controls (Control group > HD-ESRD group)

Condition	Brain regions	L/R	BA	Cluster size	Peak of coordinates (MNI)			*F -*value
					X	Y	Z	
0- back vs. 0- back	Medial frontal gyrus	R	10	25	23	52	14	23.25
	Inferior frontal gyrus	R	47	118	47	10	26	28.09
1- back vs. 1- back	Superior frontal gyrus	L	8	24	18	−19	39	32.7
		R	8	45	14	33	52	30.25
	Medial frontal gyrus	L	10	35	−28	12	58	29.98
		R	10	68	23	52	14	27.45
	Inferior frontal gyrus	R	47	160	47	10	26	25.36
	Inferior parietal lobule	R	7	47	42	42	−4	29.25
	Anterior cingulate cortex	R	24	23	−28	10	29	15.13
	Posterior cingulate cortex	L/R	29/30	45	35	−46	42	25.52
	Insula cortex	L	13	25	33	−25	14	30.71
2- back vs. 2- back	Superior frontal gyrus	L	8	78	18	−19	39	29.51
		R	8	83	14	33	52	24.34
	Middle frontal gyrus	L	10	56	−28	12	58	22.09
		R	10	112	23	52	14	28.11
	Inferior frontal gyrus	L	47	280	47	10	26	15.36
		R	47	78	−36	12	32	17.54
	Superior/Inferior parietal lobule	L	7	52	41	−30	30	31.59
		R	7	99	42	42	−4	24.93
	Anterior cingulate cortex	L/R	24	5	−1	−40	33	14.41
	Posterior cingulate cortex	L/R	29/30	60	35	−46	42	23.69
	Parahippocampal cortex	L	27/36	37	23	−38	3	26.32
		R	27/36	45	14	−33	4	27.65
	Insula cortex	L	13	60	−37	−29	−3	28.82
		R	13	47	33	−25	14	20.45
	Precuneus	R	7	79	38	−67	35	13.28

### Correlation analyses between the percentage of BOLD signal change in the frontal cortex and parietal lobule, and the laboratory results and cognitive performance in HD-ESRD patients

**Fig. 6:**
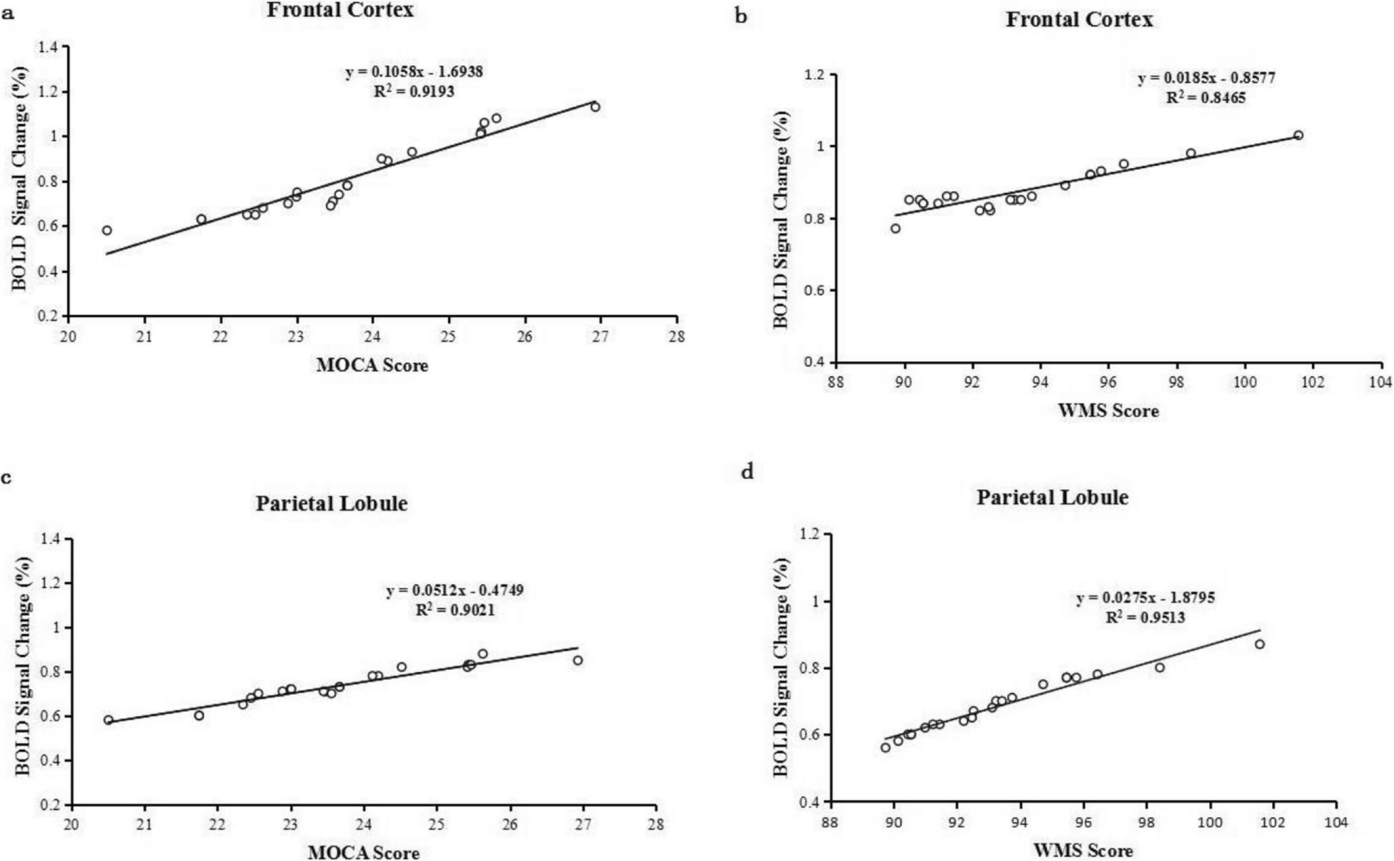
Correlation analyses between the percentage of BOLD signal change in the frontal cortex and parietal lobule and MoCA and WMS score in HD-ERSD patients. (a) MoCA score positively correlated with the percentage of BOLD signal change in the bilateral frontal cortex. (b) WMS score positively correlated with the percentage of BOLD signal change in the bilateral frontal cortex. (c) MoCA score positively correlated with the percentage of BOLD signal change in the bilateral parietal lobule. (d) WMS score positively correlated with the percentage of BOLD signal change in the bilateral parietal lobule

Pearson correlation analyses indicated that both the MoCA and WMS scores were positively correlated with the percentage of BOLD signal change in the frontal cortex, including MFG, IFG, SFG and ACC (*r* = 0.959, *P* = 0.000; *r* = 0.920, *P* = 0.000) (Fig. 6a,b), and both the MoCA and WMS scores were also positively correlated with the percentage of BOLD signal change in the parietal lobule (*r* = 0.954, *P* = 0.000; *r* = 0.975, *P* = 0.000) (Fig. 6c,d). In HD-ERSD patients, there was no correlation between the percentage of BOLD signal change and HAD-M, SAS scores in any SWM-related brain regions (all *P*s > 0.05, FDR corrected).

**Fig. 7:**
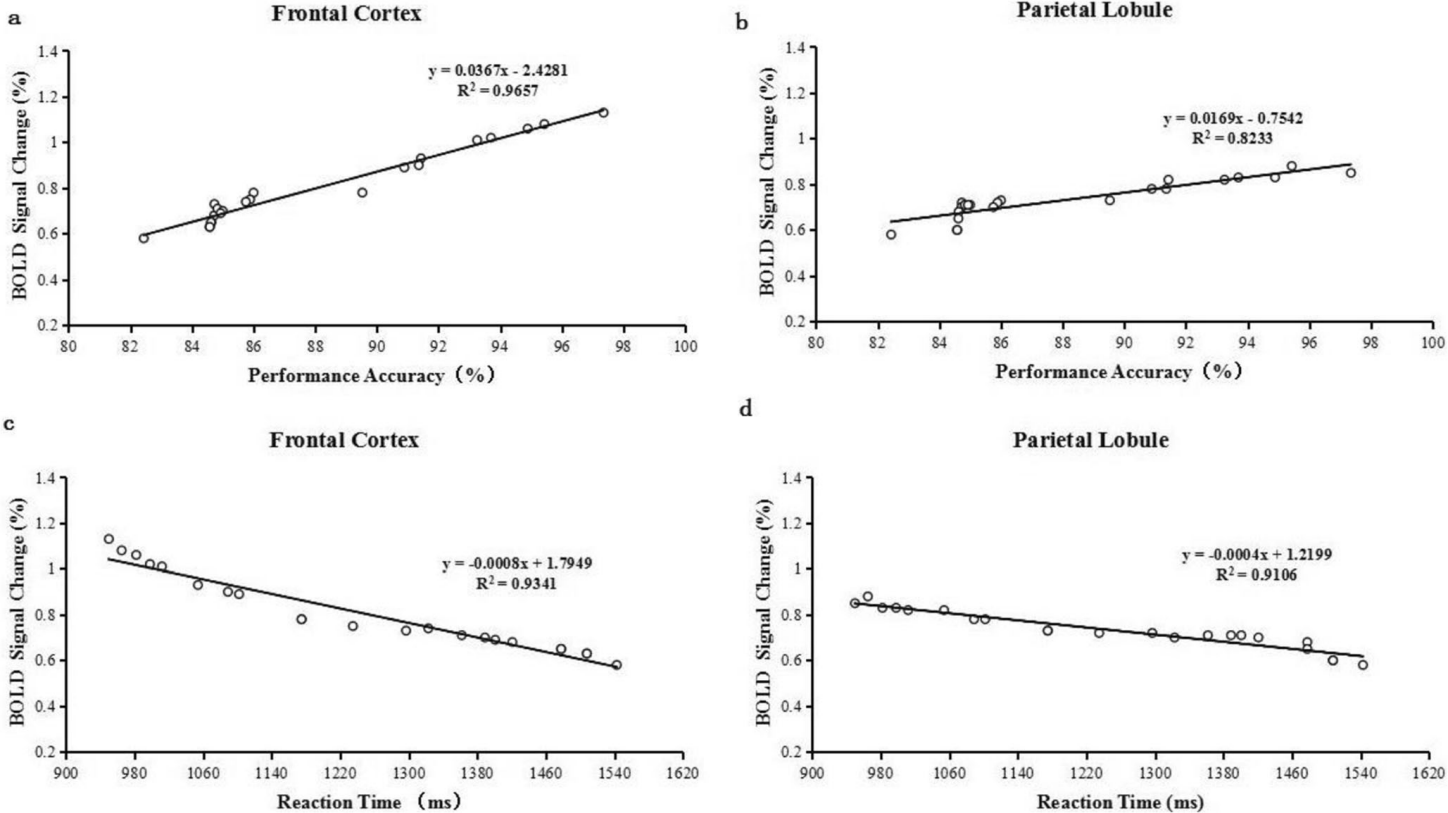
Correlation analyses between the percentage of BOLD signal change in the frontal cortex and parietal lobule and n-back task performance in HD-ERSD patients. (a) Performance accuracy positively correlated with the percentage of BOLD signal change in the bilateral frontal cortex. (b) Performance accuracy positively correlated with the percentage of BOLD signal change in the bilateral parietal lobule. (c) Reaction time negatively correlated with the percentage of BOLD signal change in the bilateral frontal cortex. (d) Reaction time negtively correlated with the of percentage BOLD signal change in the bilateral parietal lobule

Pearson correlation analyses indicated that the performance accuracy was positively correlated with the percentage of BOLD signal change both in the frontal cortex (*r* = 0.983, *P* = 0.000) and parietal lobule (*r* = 0.907, *P* = 0.000) (Fig. 7a,b); the reaction time was negatively correlated with the percentage of BOLD signal change both in the frontal cortex (*r* = −0.966, *P* = 0.000) and parietal lobule (*r* = −0.954, *P* = 0.000) (Fig. 7c,d).

Correlation results between the percentage of BOLD signal change both in brain areas and the blood biochemistry results of HD-ERSD patients showed that, both serum creatinine and urea levels were negatively correlated with the percentage of BOLD signal change both in the frontal cortex and parietal lobule (all *P*s < 0.05, FDR corrected), and both serum creatinine and urea levels were negatively correlated with the MoCA, MMSE and WMS scores (all *P*s < 0.05, FDR corrected).

BA, Brodmann area; MNI, Montreal Neurological Institute; L, left hemisphere; R, right hemisphere.

## Discussion

The MoCA, MMSE and WMS tests have been widely used in assessing cognitive and memory functions (Gong et al. 1989; Nasreddine et al. 2005; Wang et al. 2010). Block-design BOLD-fMRI was used to identify the location of impaired spatial working memory-related brain areas, leading to three main findings. First, HD-ESRD patients exhibit impaired spatial working memory abilities. Second, they show less activation in the bilateral SFG, MFG IFG, SPL/IPL and ACC, PCC and the insula when compared to controls. The performance accuracy is positively correlated with the percentage of BOLD signal change both in the frontal cortex and parietal lobule, while the reaction time is negatively correlated. Third, higher serum creatinine and urea levels might affect SWM function, involving both behavioral and brain activations in the frontal cortex and parietal lobule of HD-ESRD patients. Our current results could help to understand the neural mechanisms of SWM impairments in HD-ESRD patients, and provide neuroimaging evidence that may be used for early diagnosis and intervention in cognitive decline.

In this study, we found that the HD-ESRD patients have poorer total scores for MoCA compared with the control group, the main differences between the two groups are in abstractness and memory, visuospatial/executive function, attention, and language. Therefore, mild cognitive impairment (MCI) is speculated to occur in HD-ESRD patients according to relevant standards. WMS test indicates that HD-ESRD patients have poorer total scores compared with the control group, with significant differences between the two groups mainly related to associative learning and memory quotient. HD-ESRD patients show slightly lower MMSE scores than healthy controls, confirming that the memory of HD-ESRD patients is impaired. The above results of the MoCA, MMSE and WMS tests are consistent, these suggest that cognitive function, especially memory function, is impaired in HD-ESRD patients, which is consistent with previous studies(Bugnicourt et al. 2013; Hermann et al. 2014; Murray 2008).

In n-back task performance, we found that as the task difficulty increases, the performance accuracy presented a decreasing trend and the reaction times an increasing trend in HD-ESRD patients, thus indicating that there existe a task load effect. The HD-ESRD patient group show lower performance accuracy and longer response time under the three n-back tasks than controls, especially in the 2-back task, suggesting that HD-ESRD patients had impaired spatial working memory abilities. These results are consistent with a prior study using a similar n-back task paradigm (Owen et al. 2005). The functions involved in performing the n-back task include the storage, retelling and processing of spatial information, all of which are related to the functional activities of brain regions related to working memory. Therefore, we hypothesized that SWM impairment in HD-ESRD patients is associated with dysfunction of these brain regions.

In this study, fMRI examination revealed similar brain activity patterns during the n-back task in both HD-ESRD patients and controls, including the SFG/MFG/IFG, SPL/IPL, ACC/PCC and insula cortex, with right hemispheric dominance, consistent with brain regions previously reported in studies of working memory (Nystrom et al. 2000; Ray et al. 2008). Therefore, we defined these brain regions as regions of interest (ROIs) of SWM .

We found that the BOLD signal responses of the above SWM-related ROIs change with different n-back task loads. As the memory load increases, more brain regions are activated, and the intensity of these brain regions increases gradually, as the result of load effect (Callicott et al. 1999; Honey et al. 2002). Our study found that all ROIs presented a load effect in controls, whereas for the HD-ESRD patients, although some ROIs were activated under the three task loads, only the SFG, ACC, PCC and insula cortex exhibited task load effect, but not the MFG, IFG and SPL/IPL. In addition, those load effects were more significant under 2-back task. These results indicate that during working memory task, HD-ESRD patients with MCI and memory dysfunction exhibite loss of load effect in the frontal and parietal regions. Previous resting-state functional MRI studies indicate that ESRD patients have diffuse decreased ReHo values (Liang et al. 2013), and the ReHo values are significantly lower in HD-ESRD patients than in non-HD ESRD patients (Chen et al. 2015). However, few studies examine the effects of HD on cognitive function or the incidence of structural and functional neuroimaging abnormalities in ESRD patients, and most of these studies detected spontaneous neural activity by rs-fMRI (Prohovnik et al. 2007; Radic et al. 2011; Wolfgram et al. 2015). In one experimental study, Zhang et al. (Zhang et al. 2013) demonstrate predominantly decreased gray matter volume in the following brainareas: bilateral occipital lobes, bilateral lingual lobes, bilateral calcarine, precuneus/posterior cingulate cortex/cuneus, bilateral fusiform, right frontal lobe, bilateral superior temporal gyri, bilateral temporal pole, left hippocampus/ parahippocampus, left insula, bilateral uncus, right parahippocampus, right amygdala in ESRD patients, which is associated with neurocognitive dysfunction, and serum urea level may be a risk factor for decreased gray matter in ESRD patients. Hsieh et al. (Hsieh et al. 2009), who characterize and compare regional differences in anisotropy in patients with ESRD and in healthy controls by using diffusion-tensor imaging to understand the effect of ESRD and hemodialysis on the microstructures of white matter, reporte that the ESRD group have significantly lower FA values in all regions (including the bilateral frontal, parietal, occipital and temporal white matter, and the genu and splenium of corpus callosum) than the control group. In our study, some the fMRI deactivation regions are same to these brain structural changes in the obove studies, we also find that both serum creatinine and urea levels is negatively correlated with the percentage of BOLD signal change both in the frontal cortex and parietal lobule. It is possible that HD procedure is likely to cause these abnormalities (Bossola et al. 2011; Chen et al. 2015; Odagiri et al. 2011). HD can lead to the transfer of body fluids and electrolytes, leading to edema and lowering of cerebral blood pressure and cerebral perfusion, which may have adverse effects on the cognitive function of HD patients (Drew et al. 2013; Hata et al. 1993). Hermann et al. ‘s results suggest that HD may disrupt cerebral blood flow and oxygen saturation, leading to cognitive impairment (Hermann et al. 2014).

Our study indicates that the right MFG and IFG have lower activation in HD-ESRD patients when comparing the 0-back vs. 0-back task between the two groups (Control Group > HD-ESRD Group), whereas for 1-back vs. 1-back task, the bilateral SFG and MFG, right IFG, IPL and ACC, PCC and left insula have lower activation in HD-ESRD patients, and for 2-back vs. 2-back task, the bilateral SFG, MFG IFG, SPL/IPL and ACC, PCC and insula show less activation in the HD-ERSD patients. These results are consistent with several previous studies (Callicott et al. 2000; Perlstein et al. 2001). Thus, the load effect of a BOLD signal can be used as an effective indicator to judge whether there is working memory dysfunction. The prefrontal cortex (PFC) and ACC play an important role in working memory processing (Leung et al. 2005; Rowe et al. 2000). The PFC is considered as the consolidation and operation center of neural mechanism in working memory, and is responsible for the meta-processing of spatial position sequence, including attention and inhibition, as well as the management and integration of memory information (Funahashi 2006). The parietal lobe is the main place for storage of spatial information (Berryhill and Olson 2008). Some studies suggest that the precuneus plays a particularly important role in maintaining visual working memory and visual spatial storage (Diwadkar et al. 2000). Preclinical studies and biopsy findings suggest that the hippocampus mediates working memory (Zhang et al. 2015), which is also confirmed in our study showing that lower activation in the right precuneus and bilateral parahippocampal gyrus is associated with worse cognitive function in 2-back task.

Previous rs-fMRI studies have revealed that extensively impaired cortical and subcortical network connectivity in HD-ESRD patients is more directly associated with neuropsychological disorders. In particular, the frontal cortex is associated with cognitive and memory dysfunction (Funahashi 2006; Zheng et al. 2014). In this study, both the MoCA and WMS scores are positively correlated with the percentage of BOLD signal change in the frontal cortex and parietal lobule in HD-ESRD patients. Another previous fMRI study demonstrated that during working memory task, MCI patients exhibit reduced activation in the frontoparietal regions compared with control subjects (Saykin et al. 2004). In this study, we also observed that the performance accuracy is positively correlated with the percentage of BOLD signal change both in the frontal cortex and parietal lobule, and the reaction time is negatively correlated. More importantly, that lower activation in frontal cortex and parietal lobule is associated with worse cognitive function in the HD-ESRD patients. These results demonstrate that the abnormal brain activity patterns of frontal cortex and parietal lobule may reflect the neural mediation of SWM impairment.

There are several limitations in this study. One is that the sample size of our patient group is relatively small because our inclusion criteria and matching criteria are relatively strict for HD-ESRD patients. More samples will be collected in the further studies. The other is that, since the duration of ESRD and the duration of hemodialysis in ESRD patients may affect the results, some longitudinal fMRI studies may help to understand the progression of cognitive impairment during long-term hemodialysis.

## Conclusions

In summary, we used BOLD-fMRI technique under a block-designed n-back task to explore potential abnormal brain activity patterns of SWM in HD-ESRD patients. Our findings indicate that HD-ESRD patients have lower cognitive and memory capability, and the abnormal brain activity patterns of the frontal cortex and parietal lobule may reflect the neural mediation of SWM impairment. Thus, long-term maintenance hemodialysis may have adverse effects on cognitive and memory function in ESRD patients. In terms of treatment, clinical nursing staff should pay more attention to the cognitive impairment of the HD-ERSD patients, especially for SWM impairment, giving corresponding intervention measures to avoid the decline of spatial working memory function.
